# Hemodynamic responses in amygdala and hippocampus distinguish between aversive and neutral cues during Pavlovian fear conditioning in behaving rats

**DOI:** 10.1111/ejn.12057

**Published:** 2012-11-22

**Authors:** Stephen B McHugh, Andre Marques-Smith, Jennifer Li, J N P Rawlins, John Lowry, Michael Conway, Gary Gilmour, Mark Tricklebank, David M Bannerman

**Affiliations:** 1 Department of Experimental Psychology, University of OxfordOxford, UK; 2 Lilly Centre for Cognitive Neuroscience, Discovery Biology, Lilly Research Centre, Lilly UKWindlesham, Surrey, UK; 3 Department of Chemistry, National University of IrelandMaynooth, Co. Kildare, Ireland

**Keywords:** amygdala, extinction, fear, functional magnetic resonance imaging, hippocampus, tissue oxygen

## Abstract

Lesion and electrophysiological studies in rodents have identified the amygdala and hippocampus (HPC) as key structures for Pavlovian fear conditioning, but human functional neuroimaging studies have not consistently found activation of these structures. This could be because hemodynamic responses cannot detect the sparse neuronal activity proposed to underlie conditioned fear. Alternatively, differences in experimental design or fear levels could account for the discrepant findings between rodents and humans. To help distinguish between these alternatives, we used tissue oxygen amperometry to record hemodynamic responses from the basolateral amygdala (BLA), dorsal HPC (dHPC) and ventral HPC (vHPC) in freely-moving rats during the acquisition and extinction of conditioned fear. To enable specific comparison with human studies we used a discriminative paradigm, with one auditory cue [conditioned stimulus (CS)+] that was always followed by footshock, and another auditory cue (CS−) that was never followed by footshock. BLA tissue oxygen signals were significantly higher during CS+ than CS− trials during training and early extinction. In contrast, they were lower during CS+ than CS− trials by the end of extinction. dHPC and vHPC tissue oxygen signals were significantly lower during CS+ than CS− trials throughout extinction. Thus, hemodynamic signals in the amygdala and HPC can detect the different patterns of neuronal activity evoked by threatening vs. neutral stimuli during fear conditioning. Discrepant neuroimaging findings may be due to differences in experimental design and/or fear levels evoked in participants. Our methodology offers a way to improve translation between rodent models and human neuroimaging.

## Introduction

Understanding the neural basis of aversive learning is a fundamental goal in neuroscience, and Pavlovian fear conditioning is the dominant experimental paradigm (Davis, 1992; Phelps & LeDoux, 2005). Typically, an initially neutral cue such as a tone is paired with a painful, unconditioned stimulus (US) such as electric shock. With sufficient training, the cue becomes a conditioned stimulus (CS+) with its own affective significance. A second cue (CS−), never paired with the US, can be used to assess the specificity of the ‘CS+ → US’ association. Subsequent presentations of the CS+ without the US leads to extinction, in which a new ‘CS+ → no US’ association is formed. This simple paradigm can be used in rodents and humans in essentially the same form, and it is assumed that it engages the same brain structures.

Studies in rodents argue strongly that the amygdala and hippocampus (HPC) play key roles in fear conditioning (for reviews, see Davis, 1992; Maren, 2001; Maren & Quirk, 2004). Lesions of the basolateral (BLA) or central amygdala prevent fear conditioning to discrete cues and the training context (Iwata *et al*., 1986; LeDoux *et al*., 1990; Maren *et al*., 1996; Wilensky *et al*., 2006). HPC lesions consistently disrupt contextual conditioning (Selden *et al*., 1991; Phillips & LeDoux, 1992; Kim *et al*., 1993) and, in some circumstances, cue conditioning (Richmond *et al*., 1999; Maren & Holt, 2004; Zelikowsky *et al*., 2012). Moreover, fear conditioning increases CS+-evoked single-unit activity in amygdala and HPC neurons (Quirk *et al*., 1995; Goosens *et al*., 2003; Moita *et al*., 2003; Herry *et al*., 2008), and increases field potential amplitude in a manner congruent with the induction of long-term potentiation (McKernan & Shinnick-Gallagher, 1997; Rogan *et al*., 1997). The importance of the amygdala and HPC in rodent fear conditioning is firmly established.

However, several human functional magnetic resonance imaging (fMRI) studies report only transient amygdala or HPC activation, and some have failed to observe activation at all. Indeed, a recent meta-analysis failed to find convincing evidence of higher blood oxygen level-dependent (BOLD) signals evoked during CS+ vs. CS− trials in either structure (Mechias *et al*., 2010). Subsequently, it has been argued that hemodynamic signals are ill-suited or even unable to detect the sparse neuronal activity underlying fear conditioning (Bach *et al*., 2011). An alternative explanation, however, is that human studies may not always evoke fear levels comparable to rodent studies, and therefore may not engage the same neural circuits.

**FIG. 1:**
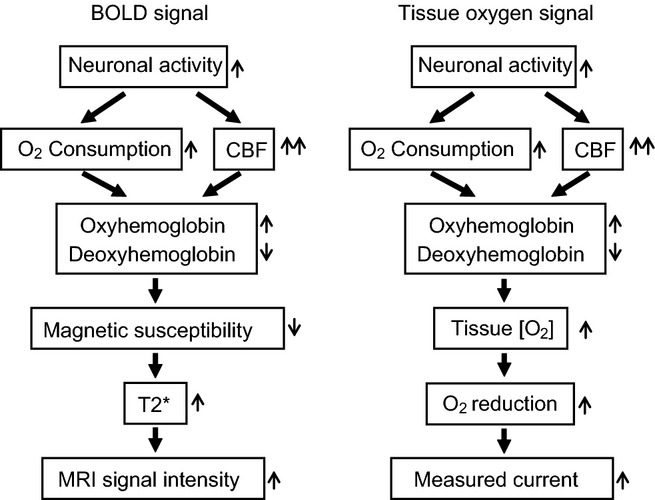
Flow chart illustrating the physiological events linking increased neuronal activity to higher blood oxygen level-dependent (BOLD) signals (left panel) and higher tissue oxygen signals (right panel). CBF, cerebral blood flow; O_2_ reduction, electrochemical reduction of O_2_ caused by applying a negative potential (−650 mV) to the sensing electrode; Tissue [O_2_], tissue oxygen concentration. The double arrow indicates a proportionally greater increase in CBF compared with O_2_ consumption, hence positive BOLD and T_O2_ signals. MRI, magnetic resonance imaging.

Here we investigated in freely-moving rats whether hemodynamic responses in the BLA, dorsal HPC (dHPC) and ventral HPC (vHPC) could distinguish between threat-predicting (CS+) and neutral (CS−) cues. We recorded hemodynamic responses in the form of tissue oxygen (T_O2_) signals, which are closely related to the BOLD signal and are dependent upon the same physiological mechanisms (Fig. 1; Thompson *et al*., 2003; Offenhauser *et al*., 2005; Logothetis, 2007; Lowry *et al*., 2010; Li *et al*., 2011; McHugh *et al*., 2011). CS+ and CS−-evoked T_O2_ signals were recorded during pre-exposure, training and extinction of conditioned fear.

## Materials and methods

### Subjects

This study used eight naïve male Sprague–Dawley rats (Harlan Olac, Bicester, UK), approximately 3 months old at the start of the experiment, and housed in a temperature- and humidity-controlled room under a 12 h light : dark cycle (lights on 07:00–19:00 h). Testing took place during the light cycle. Rats were housed four per cage before surgery, singly for 1 week after surgery, and two per cage thereafter, with *ad libitum* food and water. The experiments were conducted in accordance with the United Kingdom Animals Scientific Procedures Act (1986) under project license PPL 30/1989, and were approved by local ethical review for the University of Oxford.

### Electrode construction

T_O2_ was recorded amperometrically using carbon paste electrodes (CPEs), as described previously (McHugh *et al*., 2011). CPEs were constructed from 8T Teflon®-insulated silver wire (200 μm bare diameter, 270 μm coated diameter; Advent Research Materials, Suffolk, UK) soldered into gold-plated socket contacts (E363/0, Plastics One, Roanoke, VA, USA). The Teflon® insulation was slid along the wire to create a ∼2-mm-deep cavity, which was packed with carbon paste (2.8 g carbon powder; Sigma-Aldrich, St Louis, MO, USA; Catalogue No. 282863, mixed with 1.0 mL of silicone oil; Sigma-Aldrich; Catalogue No. 17563-3). The Teflon® insulation on the CPEs was flush with the compacted and smoothed carbon paste such that the active part of the electrode was a flat disk with diameter 200 μm (area: 0.03 mm^2^). Reference and auxiliary electrodes were made from 8T Teflon®-insulated silver wire, with one end soldered into a gold-plated contact (E363/0, Plastics One).

### Surgery

Rats were anesthetized with Isoflurane (3–4% in 4 L/min O_2_ for induction, 1.5–2% in 1 L/min O_2_ for maintenance) and placed in a stereotaxic frame with the head level between bregma and lambda. Body temperature was maintained at 37 °C via a homoeothermic blanket connected to a rectal thermometer (Harvard Apparatus, MA, USA). Lignocaine (1%) was topically applied to the skin, and Viscotears® (Novartis Pharma AG, Basel, Switzerland) was used to prevent corneal drying. An incision was made along the midline and the periosteum retracted. Skull holes were drilled to allow the insertion of electrodes and skull screws. Rats were implanted with CPEs into the BLA [anterior–posterior (A–P): −2.8; medial–lateral (M–L): +5.0; dorsal–ventral (D–V): −7.0], dHPC (A–P: −3.6; M–L: +2.2; D–V: −3.2) and vHPC (A–P: −5.3; M–L: −4.7; D–V: −6.5). Reference and auxiliary electrodes were implanted into the cortex. The gold contacts from each electrode were inserted into a six-channel electrode pedestal (MS363, Plastics One), which was secured to the skull with dental cement. Analgesia (Meloxicam, ∼2 mg/kg; s.c.) was given before and after surgery.

### Amperometric techniques

T_O2_ was measured using constant potential amperometry. A constant potential (−650 mV relative to a reference electrode) was applied to the CPEs using a low-noise potentiostat (‘Biostat’, ACM Instruments, Cumbria, UK). The applied potential produces the electrochemical reduction of dissolved O_2_ on the surface of the CPEs, inducing an electrical current, which was measured by the potentiostat. The availability of O_2_ for this two-step reaction (O_2_ + 2H^+^ + 2e^−^ → H_2_O_2_; H_2_O_2_ + 2H^+^ + 2e^−^ → 2H_2_O) is determined by the local T_O2_ concentration. Thus, changes in O_2_ concentration around the tip of the CPE produce directly proportional changes in the measured Faradaic current (Hitchman, 1978). The spatial resolution is sufficient to detect T_O2_ differences between lamina in rat whisker barrel cortex, i.e. approximately ∼400 μm (Li *et al*., 2011). Accordingly, the area of sensitivity is estimated to be a sphere with diameter twice the electrode surface diameter, i.e. a 200-μm-diameter electrode has ∼400-μm-diameter sphere of sensitivity (see also Thompson *et al*., 2003).

### Data recording

Rats were connected to the potentiostat via a six-channel commutator (SL6C, Plastics One) using screened cables (363-363 6TCM, Plastics One). A Powerlab® 8/30 (AD Instruments, Oxon, UK) was used for analog/digital conversion, and data were collected on a Windows PC running Chart® v5 software (AD Instruments). T_O2_ data were sampled continuously at 4 kHz.

### Apparatus

The pre-exposure and training sessions were conducted in an operant chamber (ENV-008-CT; Med Associates, Lafayette, IN, USA), illuminated by a ceiling-mounted house-light (ENV-215M; Med Associates). The grid floor (ENV-005) of the chamber had 18 stainless-steel rods connected to a shock generator (ENV-414S). A speaker (ENV-224AM) and a mechanical clicker device (ENV-135M) were mounted on the same wall of the chamber, at the same height. A counter-weighted arm (PHM-110P1) attached to the rear wall of the operant chamber held a swiveling commutator (SL6C, Plastics One) that allowed free movement of the tethered rat. The operant chamber was placed inside a sound-attenuating melamine cubicle (ENV-018MD). A CCD video camera with a wide-angle lens (VPC-465B, CES AG, Zurich, Switzerland) provided a full view of the interior of the chamber. Tissue paper was used to line the waste pan and this was replaced for each rat.

The extinction session took place in a novel context – a transparent plastic box (220 mm wide, 200 mm deep, 250 mm high) placed inside a sound-attenuating melamine chamber (350 mm wide, 395 mm deep, 475 mm high). The clicker and speaker were mounted on the same wall of the box, at the same height. An infra-red video camera (Maplin Electronics, UK) was mounted above the box, providing a full view of the interior. The discriminability between the novel and training contexts was enhanced in three ways. First, the floor of the novel context was solid plastic and lined with tissue paper, providing a strong tactile contrast with the metallic grid floor of the training context. Second, the novel context was not illuminated, whereas the house light was on in the training context. Third, the novel context was given a distinct odor by placing one drop of sandalwood oil (Home Fragrance Essential Oils, Body Shop, UK) onto fresh tissue paper before each session began.

CS and US delivery were controlled by a script written in med-pc iv software (Med Associates). In each session, the rat heard five tones (2900 Hz, 87 dB, 30-s duration) and five clickers (10 Hz, 87 dB, 30-s duration) in random order, but with no more than two consecutive presentations of the same stimulus. A relatively long CS duration (30 s) was chosen so that the hemodynamic response evoked by the CS could fully evolve before the arrival of the US. Note that during extinction, CS+1 preceded CS−1 for 50% of the rats, and vice versa for the other 50%. The interval between CS presentations was randomly varied between 70 and 110 s (mean: 90 s; SD: 14.9 s). The US consisted of a scrambled footshock (0.5 mA, 0.5 s duration), delivered through the bars of the grid floor, that co-terminated with offset of the CS+ (i.e. US onset at 29.5 s after CS onset). The script controlled the delivery of transistor-transistor-logic pulses to the Powerlab 8/30 ADC to ensure that CS and US delivery were accurately synchronized to T_O2_ data acquisition (1-ms resolution).

### Procedure

The aim was to adopt a fear-conditioning paradigm that resembled those used in human fMRI studies in order to be able to compare the T_O2_ signals with BOLD responses associated with fear-inducing stimuli. Specifically, this involved using a discriminative fear-conditioning paradigm in which a CS+ (tone or clicker, counterbalanced) was paired with an aversive US (footshock); whereas a CS− (clicker if tone was CS+, tone if clicker was CS+) was never paired with footshock. Thus, T_O2_ signals evoked by the CS+ could be compared directly with signals evoked by the CS−, allowing for a CS+ vs. CS− contrast as would be used in BOLD fMRI. Fear conditioning began 3 weeks after surgery, and the experimental design is shown in Table 1.

**Table 1:** Summary of experimental design

Day		Trials
1	Pre-exposure (training context)	5 × tone, 5 × clicker (no shocks to either)
2	Training I (trials 1–5)	5 × CS− → no shock, 5 × CS+ → shock
3	Training II (trials 6–10)	5 × CS− → no shock, 5 × CS+ → shock
4	Extinction I (novel context)	5 × CS−, 5 × CS+ (no shocks to either)

Rats were first pre-exposed to the tone and clicker without any footshocks (Day 1: 5 × tone, 5 × clicker). This pre-exposure phase was included because pre-exposure to the CS+ and CS− are commonly used in both human fMRI (Buchel *et al*., 1998, 1999; LaBar *et al*., 1998) and rodent electrophysiology experiments (Goosens *et al*., 2003; Moita *et al*., 2003; Herry *et al*., 2008). After the pre-exposure session, the rats then received 10 training trials over 2 days in which either the tone or clicker was always followed by footshock (Days 2 and 3: 5 × CS−, 5 × CS+ footshock, per day). Rats were conditioned over 2 days because pilot testing revealed that one session was insufficient to see robust CS+/CS− discrimination. Training was followed by extinction in a novel context during which no shocks were given after either the CS+ or CS− (Day 4: 5 × CS−, 5 × CS+; no shocks).

Apart from the presence or absence of shocks, the testing procedure on each day was otherwise identical. The rat was taken to the testing room in a transport cage, connected to the recording equipment, and placed into the conditioning chamber (Days 1–3) or the novel context (Day 4). The potential was then applied to the electrodes for 15 min to ensure a stable T_O2_ signal. Then the 22-min experimental session began (total time in box per day: 37 min). At the end of each session, the rat was removed from the chamber, disconnected from the equipment and returned to the holding room. The chamber was cleaned with a solution of 10% (v/v) ethanol in water, and the napkin lining the waste pan (training context) or lining the floor (novel context) was replaced before the next session began.

### Data analyses

Freezing behavior was measured using a script in NIH Image (NIH, Bethesda, MD, USA). The script collected one frame of video per second and compared consecutive frames for changes in pixels. If a sufficient proportion of pixels changed then the rat was adjudged to be moving; if not, then the rat was adjudged to be freezing. The script was calibrated so that pixel changes caused by random camera noise and breathing movements were not sufficient to signal a movement score. This automated system has over 80% concordance with human ratings of freezing behavior. A detailed description can be found in Richmond *et al*. (1998). The counts of freezing for each 30-s CS presentation were then expressed as a percentage. For example, a rat freezing for 18 s during the 30-s CS would receive a freezing score of 60%. For correlational analyses with T_O2_ responses, we also calculated the freezing difference score by subtracting the mean CS−-evoked freezing from the mean CS+-evoked freezing.

T_O2_ data were down-sampled from 4 kHz to 10 Hz, and the signal change from baseline (ΔT_O2_ signal) was calculated by subtracting the mean T_O2_ signal during the 5 s before CS onset (i.e. baseline) from the T_O2_ signal during the 30-s CS presentation. Then, the 30-s signal was divided into 15 × 2-s timebins (i.e. 0–2 s, 2–4 s, 4–6 s, …, 28–30 s), with each data point equal to the mean value during each 2-s timebin. To investigate the correlation with behavior, we also calculated a T_O2_ difference score by subtracting the mean CS−-evoked T_O2_ signal from the mean CS+-evoked T_O2_ signal. Difference scores were calculated separately for each rat and for each brain region for the second training day and the extinction day.

### Statistical procedures

Data were analysed using *t*-tests, analysis of variance (anova) and Pearson correlation in spss (version 15; SPSS, IL, USA). anovas are described using a modified version of Keppel's (1982) notation in which the dependent variable is defined in the form: *A*_2_ × *B*_3_ × *S*_8_, where *A* is a factor with two levels, *B* a factor with three levels, and *S*_8_ denotes that eight subjects were included in the analysis.

For each day, we calculated the mean freezing and T_O2_ responses for the five CS+ vs. the five CS− trials. T_O2_ responses were analysed separately for each brain region (BLA, vHPC, dHPC) using repeated-measures anova, with CS type (CS+ vs. CS−) and timebin (1–15, covering the 30-s CS duration in 2-s timebins) as within-subject factors [i.e. CS type_2 (CS+,CS−)_ × timebin_15_ × *S*_8_]. For the extinction session, we also investigated responses to the first and the last CS+ and CS− trials of the session: i.e. CS−1, CS+1, CS−5, CS+5. The rationale for this approach was that responses to CS+1 (vs. CS−1) reflect the pure expression of conditioned fear, which is logically unconfounded by any extinction process whereas, comparatively, responses to CS+5 (vs. CS−5) reflect, in part, the consequences of extinction, i.e. learning that the CS+ is no longer followed by shock. These extinction session data were analysed using the following models, for freezing: CS type_2 (CS+,CS−)_ × trial number_2 (CS1,CS5)_ × *S*_8_; and for T_O2_: CS type_2 (CS+,CS−)_ × trial number_2 (CS1,CS5)_ × timebin_15_ × *S*_8_. Separate analyses for CS+ and CS− stimuli, including all five trials of the extinction session, were also conducted. For all analyses, interactions were investigated using simple main effects (SME) and pairwise comparisons with the familywise error rate at *α* = 0.05. Pairwise comparisons for CS type × timebin interactions (e.g. for CS+ vs. CS−) are reported as time periods after stimulus onset (e.g. higher CS+ vs. CS− signals during timebins 4, 5 and 6 are reported as: CS+ > CS−, 6–12 s, *P <* 0.05). Figures show the mean ± 1 standard error of the mean (SEM) for CS+- and CS−-evoked responses (upper panels), and the difference contrast (CS+ > CS−) ± 1 standard error of the difference (lower panels). Note that negative values for the difference contrast indicate CS− > CS+. We found no evidence of a CS+ vs. CS− contrast in either the behavioral or T_O2_ data on the first day of training, and so only data from training Day 2 are shown in the main figures. Data from training Day 1 are shown in Fig. S1.

### Histology

At the end of the experiments, rats were injected with Euthatal (200 mg/mL sodium pentobarbitone; 200 mg/kg i.p.) and perfused transcardially with physiological saline (0.9% NaCl) followed by 10% formol saline (10% formalin in physiological saline). The brains were then removed and placed in 10% formol saline. The brains were then transferred to 30% sucrose–formalin and frozen. Coronal sections (50 μm) were cut on a freezing microtome and stained with Cresyl violet to enable visualisation of the electrode placements.

## Results

### Histology

**FIG. 2:**
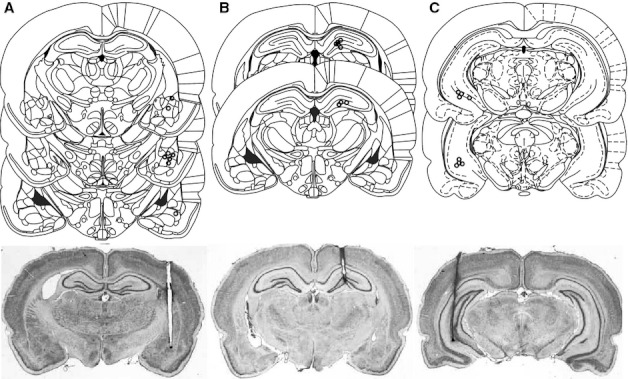
Reconstructions and representative photomicrographs of electrode positions in (A) BLA, (B) dHPC and (C) vHPC. Reconstructions are based on the atlas of Paxinos & Watson (1998).

CPEs were successfully targeted to the BLA, dHPC and vHPC in every rat, as shown in Fig. 2. There were no systematic differences in T_O2_ signals based on exact placement within the BLA or subfield placements within dHPC or vHPC. No rats were excluded on histological grounds.

### Behavior

**FIG. 3:**
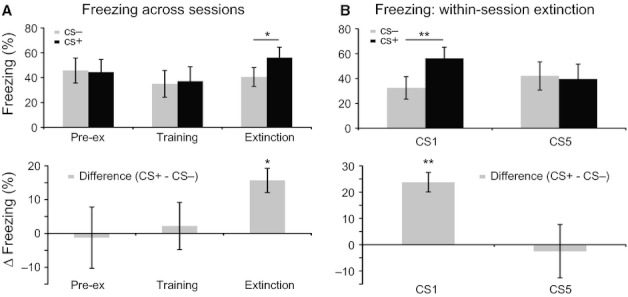
Freezing behavior. (A) Upper panel: percentage (%) freezing (±SEM) evoked by the CS− (gray) and CS+ (black) during pre-exposure, Day 2 of training, and extinction. Lower panel: the difference contrast (CS+ minus CS− ± standard error of the difference, SED) for the data shown in the upper panel. (B) Upper panel: % freezing (±SEM) evoked by the CS−1 and CS+1 (left) and CS−5 and CS+5 (right) during extinction (CS−, gray; CS+, black). Lower panel: the difference contrast (±SED) for the freezing data shown in the upper panel. Pre-ex, pre-exposure session. *P < 0.01; **P < 0.001. CS, conditioned stimulus.

During the pre-exposure session, rats were initially active during CS presentations (i.e. they exhibited low levels of freezing), but became less active towards the end of the session, presumably reflecting habituation to the conditioning chamber. Importantly, behavioral responses to the CS+ and CS− were almost identical during the pre-exposure session (Fig. 3A).

Somewhat surprisingly, during the training sessions, there was no significant behavioral discrimination, perhaps because discriminative freezing was masked by contextual conditioning and/or the unconditioned response to the shock. Analysis of freezing responses during the 10 CS+ vs. 10 CS− training trials (anova: CS type_2_ × trial number_10_ × *S*_8_) revealed no effect of CS type, trial number or interaction (all *F* < 1.2; *P* > 0.3; see Fig. S2).

However, even though rats did not discriminate during training, they did learn to discriminate between the CS+ and CS−, and during the extinction session they froze significantly more during CS+ than CS− trials. Analysis of CS+ and CS− evoked freezing over pre-exposure, training and extinction (anova: day_3_ × CS type_2_ × *S*_8_) revealed a significant CS type × day interaction (*F*_2,14_ = 3.9; *P =* 0.045), with higher freezing during CS+ than CS− trials on the extinction day (*P =* 0.003) but not during pre-exposure or training (both *P >* 0.8). Thus, during the extinction session, the rats demonstrated clear behavioral discrimination between the CS+ and CS−.

The previous analysis of the extinction day used the averaged freezing response for the five CS+ vs. the five CS− presentations. To dissociate fear expression from fear extinction, we also investigated responses to the first and last CS+ and CS− trials of the extinction session [i.e. (CS+1 vs. CS−1) vs. (CS+5 vs. CS−5)], using the rationale that rats would expect footshock following CS+1 but, after four non-reinforced trials, would be less likely to expect shock following CS+5. Consistent with this prediction, all eight rats froze more during CS+1 than CS−1, but only two out of eight rats froze more during CS+5 than CS−5 (Fig. 3B). Analysis of these data (anova: CS type_2 ×_ trial number_2_ × *S*_8_) revealed a significant CS type × trial number interaction (*F*_1,7_ = 7.6; *P =* 0.03), with higher freezing during CS+1 than CS−1 (*F*_1,7_ = 41.3; *P <* 0.001) but no difference between CS+5 and CS−5 (*F*_1,7_ = 0.1; *P >* 0.9). Thus, rats froze more during the CS+ than the CS− at the start of the session but not at the end, indicating that the CS+ was no longer treated as a threat stimulus. Furthermore, separate one-way anovas, conducted across all five trials of the extinction session, revealed a significant main effect of trial number for the CS+ trials (*F*_4,28_ = 3.0; *P =* 0.03), which reflected the general decrease in levels of freezing across the extinction session, but no significant main effect of trial number for the CS− trials (*F*_4,28_ < 1; *P =* 0.4).

### BLA T_O2_ signals respond to changes in ‘CS+ → US’ contingency

**FIG. 4:**
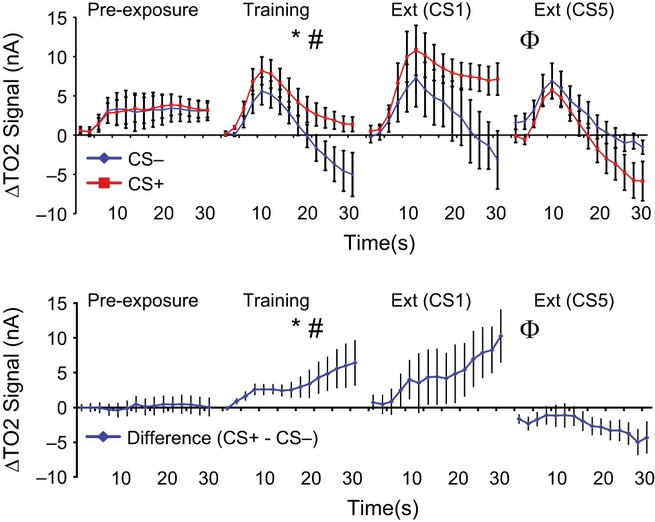
BLA tissue oxygen (T_O2_) signals. Upper panel: BLA T_O2_ signals (±SEM) evoked by the CS− (blue) and CS+ (red) during pre-exposure, Day 2 of training, and the first (CS1) and last (CS5) CS− and CS+ trials of extinction. Lower panel: the difference contrast (CS+ minus CS− ± SED) for the BLA data shown in the upper panel. *Main effect of CS, *P <* 0.05; ^#^CS type × timebin interaction, *P <* 0.05; ^Φ^CS type × trial number × timebin interaction, *P <* 0.05. CS, conditioned stimulus.

During the pre-exposure session, stimulus onset evoked a small-amplitude, positive-going T_O2_ response in the BLA to both stimuli (Fig. 4). There were no differences in T_O2_ responses to the ‘to-be-allocated’ CS+ and CS− (no effect of CS type or CS type × timebin interaction: all *F* < 0.2; *P >* 0.9). Training led to a marked change in the amplitude and shape of the CS+- and CS−-evoked T_O2_ signals (see Figs 4 and S1) and, on the second training day, CS+-evoked signals were significantly higher than CS−-evoked signals (main effect of CS type: *F*_1,7_ = 6.9; *P =* 0.03; CS type × timebin interaction: *F*_14,98_ = 2.0; *P =* 0.03; SME: CS+ > CS−, 2–16 s, *P <* 0.05; 20–30 s, 0.05 < *P <* 0.1). Thus, during training Day 2, BLA T_O2_ signals were significantly higher during CS+ than CS− trials, and this was found in the absence of significant behavioral discrimination.

### BLA T_O2_ signal correlates with behavioral discrimination during training

**FIG. 5:**
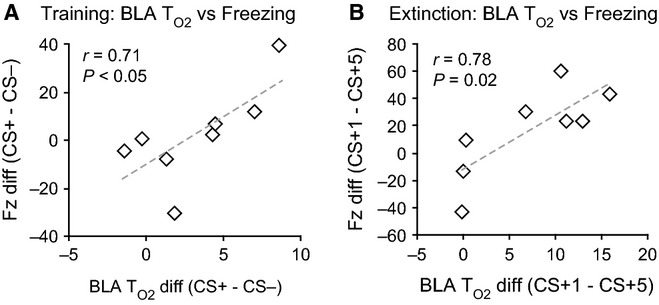
Correlation between basolateral amygdala (BLA) T_O2_ signals and freezing behavior. (A) The graph shows BLA T_O2_ signal difference contrast (CS+ minus CS−) vs. freezing difference contrast (CS+ minus CS−) for Day 2 of training. Rats that had higher CS+ than CS− T_O2_ signals exhibited higher freezing to the CS+ than CS−. (B) The BLA T_O2_ signal difference contrast (CS+1−CS+5) vs. the corresponding freezing difference contrast (CS+1−CS+5) during extinction. Larger decreases in BLA T_O2_ signal from CS+1 to CS+5 were associated with larger decreases in freezing behavior from CS+1 to CS+5. In both graphs, each data point represents the T_O2_ vs. freezing difference score for individual rats (*n* = 8). CS, conditioned stimulus.

Next, we investigated the relationship between BLA T_O2_ signals and freezing behavior. We calculated a T_O2_ difference score based on the CS+ > CS− contrast from human fMRI studies (i.e. CS+-evoked T_O2_ signal minus CS−-evoked T_O2_ signal) for each rat and plotted this against the corresponding freezing difference score (i.e. CS+-evoked freezing minus CS−-evoked freezing). Thus, each data point in Fig. 5 represents the BLA T_O2_ difference score vs. the freezing difference score for each rat (*n* = 8). BLA T_O2_ difference scores were significantly correlated with freezing difference scores during training (*r* = 0.7; *t*_6_ = 2.5, *P <* 0.05; Fig. 5A). Thus, although at a group level freezing was not significantly higher during training for CS+ than CS− trials, the BLA T_O2_ signal contrast was significantly correlated with the magnitude of behavioral discrimination.

### BLA T_O2_ responses change to reflect ‘CS+ → no US’ association during extinction in a novel context

During the extinction session, BLA T_O2_ responses averaged over the five CS+ vs. the five CS− trials were not statistically different (*F* < 1.1; *P >* 0.3; see Fig. S1). However, as illustrated in Fig. 4, BLA T_O2_ responses were markedly different at the start compared with the end of the extinction session [i.e. (CS+1 vs. CS−1) vs. (CS+5 vs. CS−5)]. BLA T_O2_ signals were higher during CS+1 than CS−1, but were actually lower during CS+5 than CS−5. Analysis of these BLA T_O2_ responses revealed a significant three-way interaction between CS type, trial number and timebin (*F*_14,98_ = 5.1; *P =* 0.001). Dissection of this three-way interaction using analysis of SME revealed three key findings.

First, BLA T_O2_ signals were significantly higher during CS+1 vs. CS−1 of extinction (CS type × timebin interaction: *F*_14,98_ = 3.9; *P =* 0.001; CS+1 > CS−1, 26–30 s, *P <* 0.05), consistent with what was seen behaviorally and confirming that the higher CS+-evoked T_O2_ signals seen during training were also present at the start of extinction.

Second, T_O2_ signals were significantly higher during CS+1 than CS+5 (trial number × timebin interaction: *F*_14,98_ = 10.0; *P =* 0.02; CS+1 > CS+5, 16–30 s, *P <* 0.05), but not for CS−1 vs. CS−5 (no effect of trial number or trial number × timebin interaction, all *F* < 1.2; *P >* 0.3), demonstrating extinction specifically for the CS+-evoked signals.

Third, T_O2_ signals were actually significantly lower during CS+5 than CS−5 (CS type × timebin interaction: *F*_14,98_ = 2.3; *P =* 0.01; CS−5 > CS+5, 0–4 s and 18–28 s, *P <* 0.05). Thus, at the start of extinction T_O2_ signals were significantly higher during CS+ than CS− trials, but by the end of extinction they were significantly higher during CS− than CS+ trials. This is consistent with the idea that the ‘CS+ → no US’ association formed during extinction leads to inhibition of the CS+-evoked BLA T_O2_ signal.

### BLA T_O2_ signals correlate with the reduction in freezing during extinction

Next, we investigated the relationship between CS+-evoked BLA T_O2_ signals and freezing behavior specifically during extinction. Six out of eights rats froze more during CS+1 than CS+5, indicative of extinction. In contrast, two out of eight rats did not exhibit reduced freezing from CS+1 to CS+5. Interestingly, in these two rats there was very little difference (or a negative difference) in the BLA T_O2_ signal contrast between CS+1 and CS+5 (i.e. CS+1 minus CS+5). In other words, like the freezing behavior, the BLA T_O2_ signals were as high (or higher) during CS+5 than CS+1 in these two rats. Conversely, in the six rats that froze more during CS+1 than CS+5, the corresponding BLA T_O2_ signals were higher during CS+1 than CS+5. To analyse these data, we calculated difference scores for freezing behavior (CS+1-evoked freezing minus CS+5-evoked freezing) and BLA T_O2_ signals (CS+1-evoked T_O2_ signals minus CS+5-evoked T_O2_ signals), and plotted these against each other (Fig. 5B). The difference between CS+1- and CS+5-evoked BLA T_O2_ signals was highly correlated with the decrease in freezing from CS+1 to CS+5 (*r* = 0.8; *t*_7_ = 3.1, *P =* 0.02). In other words, the decrease in the T_O2_ signal from CS+1 to CS+5 predicted the reduction in freezing behavior seen across the extinction session.

### HPC T_O2_ signals are higher during CS+ than CS− trials during acquisition

**FIG. 6:**
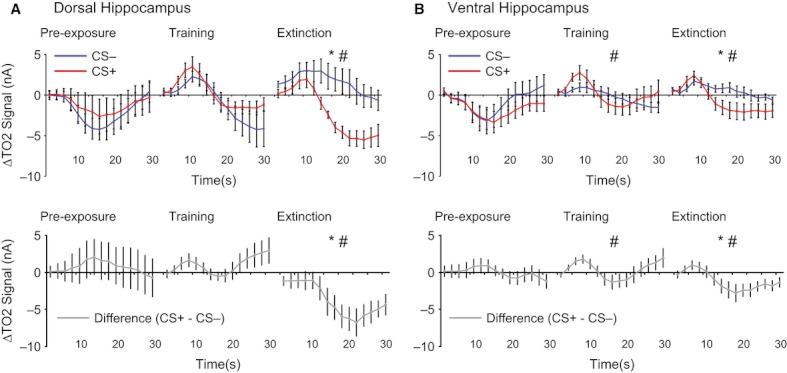
HPC T_O2_ signals. (A) Upper panel: dHPC T_O2_ signals (±SEM) evoked by the CS− (blue) and CS+ (red) during pre-exposure, Day 2 of training, and extinction. Lower panel: the difference contrast (CS+ minus CS− ± SED) for the dHPC data shown in the upper panel. (B) Upper panel: as in (A), but for vHPC T_O2_ signals. Lower panel: the difference contrast (CS+ minus CS− ± SED) for the vHPC data shown in the upper panel. *Main effect of CS, *P <* 0.05; ^#^CS type × timebin interaction, *P <* 0.01. CS, conditioned stimulus.

During pre-exposure, CS+- and CS−-evoked T_O2_ signals in dHPC and vHPC were different to those seen in the BLA, with CS onset leading to negative-going signals in both HPC subregions (compare leftmost traces in Fig. 4 vs. Fig. 6A and B). Importantly, there were no differences between the ‘to-be-allocated’ CS+- and CS−-evoked signals in either dHPC or vHPC during pre-exposure (no effect of CS type or CS type × timebin interaction: all *F* < 1.2; *P >* 0.3). However, as with the corresponding amygdala T_O2_ responses, training changed the direction and shape of responses in both HPC regions and, by the second training day, CS+-evoked vHPC T_O2_ signals were significantly higher than CS−-evoked signals (CS type × timebin interaction: *F*_14,98_ = 5.1; *P =* 0.001; CS+ > CS−, 4–8 s, *P <* 0.05). This did not reach significance in dHPC, but there was a trend toward higher CS+-evoked signals (main effect of CS type: *F*_1,7_ = 3.9; *P =* 0.09; no interaction: *F*_14,98_ = 1.3; *P >* 0.2). Thus, like the BLA, vHPC T_O2_ signals during training were higher during CS+ than CS− trials. However, unlike the BLA, neither vHPC nor dHPC difference signals (i.e. the CS+- minus CS−-evoked T_O2_ signal) were significantly correlated with the corresponding freezing difference scores during training (dHPC: *r* = 0.59, *P >* 0.1; vHPC: *r* = −0.57, *P >* 0.1).

### dHPC and vHPC T_O2_ signals are lower during CS+ than CS− trials during extinction

During extinction, dHPC and vHPC T_O2_ responses were again markedly different from those seen in the BLA (see Figs 6 and S1). As shown in Fig. 6, CS+-evoked T_O2_ responses in both dHPC and vHPC decreased below baseline after 12–14 s and were lower (on average) than CS−-evoked responses. Indeed, mean dHPC T_O2_ signals (averaged over the five CS+ vs. the five CS− trials of the session) were significantly lower during CS+ than CS− trials (main effect of CS type: *F*_1,7_ = 10.5; *P =* 0.01; CS type × timebin interaction: *F*_14,98_ = 7.8; *P =* 0.001; CS− > CS+, 14–30 s, *P <* 0.05). The same pattern of lower CS+-evoked signals was seen in vHPC (main effect of CS type: *F*_1,7_ = 5.9; *P =* 0.045; CS type × timebin interaction: *F*_14,98_ = 4.1; *P =* 0.001; CS− > CS+, 16–28 s, *P <* 0.05). Note that unlike signals in the BLA, there was no pronounced change in response from CS+1 to CS+5 in the HPC signals across the extinction session, so it is unlikely that the lower CS+- than CS−-evoked responses were a consequence of extinction (see Fig. S1). Nevertheless, both dHPC and vHPC T_O2_ signals clearly discriminated between the discrete auditory stimuli (CS+ and CS−) during the extinction session.

## Discussion

### Summary of results

Behavioral responses and T_O2_ signals from the BLA, dHPC and vHPC were recorded simultaneously in freely-moving rats during the acquisition, expression and extinction of conditioned fear. Rats froze more during CS+ than CS− trials during the extinction session in a novel context but not during training. In the BLA, T_O2_ signals were higher during CS+ than CS− trials during training and at the start of extinction, but were significantly lower during CS+ than CS− trials by the end of extinction, a pattern that may reflect the expression of the ‘CS+ → no US’ association learned during extinction. Moreover, during training, the levels of CS+/CS− discrimination present in the BLA T_O2_ signals were strongly correlated with behavioral discrimination, even though at a group level the rats did not freeze significantly more during CS+ than CS− trials. Also, during extinction, the decrease in the BLA T_O2_ signal from CS+1 to CS+5 was significantly correlated with the decrease in freezing responses from CS+1 to CS+5. T_O2_ signals in dHPC and vHPC also discriminated between the CS+ and CS−, with higher CS+-evoked signals in vHPC during training and lower CS+-evoked signals in vHPC and dHPC during extinction. Thus, in rats, hemodynamic responses in the BLA and HPC can detect the distinct patterns of neuronal activity evoked by aversive vs. neutral cues.

### Role of the amygdala in fear conditioning: from rodent studies to human neuroimaging

Rodent lesion and electrophysiological studies demonstrate an essential role for the BLA in fear conditioning and extinction (LeDoux *et al*., 1990; Maren *et al*., 1996; Herry *et al*., 2008; Sierra-Mercado *et al*., 2011), and several authors have proposed the BLA as the critical locus for CS+ → US associations (Davis, 1992; Romanski *et al*., 1993; Fanselow & LeDoux, 1999; Maren, 2001). Overall, in rodents the evidence for the role of the BLA in fear conditioning is compelling.

Given this evidence, the failure of many human fMRI fear studies to detect differential amygdala activation is surprising (Mechias *et al*., 2010). However, the interpretation that group-averaged hemodynamic signals are insensitive to the differential patterns of neural activity elicited by the CS+ vs. CS− (for discussion, see Bach *et al*., 2011) is not consistent with the present data. Even with a relatively small sample, we found that 10 training trials were sufficient to produce robust CS+/CS− discrimination in the BLA T_O2_ signal. So, at least in rats, amygdala hemodynamic responses can discriminate between aversive and neutral stimuli.

Nevertheless, the question remains as to why some fMRI studies do not observe differential amygdala activity. First, it is important to emphasize that many studies have reported robust differential amygdala activity (Morris *et al*., 1998, 2001; Armony & Dolan, 2002; Tabbert *et al*., 2005, 2006; Knight *et al*., 2009). Thus, our data are consistent with a subset of the fMRI literature. Importantly, our study shares certain design features with some of the studies cited above (Tabbert *et al*., 2005, 2006; Knight *et al*., 2009) that are not found in the majority of the studies discussed by Mechias *et al*. (2010) and Bach *et al*. (2011). First, unambiguously aversive USs were used (i.e. shock or 100 dB white noise). Second, 100% reinforcement between the CS+ and US was used (i.e. the CS+ was always followed by the US during training), not partial reinforcement. This may be particularly important as amygdala activation increases as a function of the probability of reinforcement (Dunsmoor *et al*., 2007), and the majority of human fear-conditioning studies have used partial reinforcement to avoid conflation between CS+- and US-evoked responses. These design features may be necessary, although perhaps not sufficient, to evoke differential amygdala responses to the CS+ vs. the CS−.

A further consideration concerns the levels of fear evoked in participants. Humans are aware that they will not come to any real harm during the experiment, which presumably limits their subjective experience of fear during scanning. Although we cannot know for sure, it is a reasonable assumption that rodents experience higher levels of fear than humans during fear-conditioning experiments. Equating levels of fear across species may not be possible, but a recent human fMRI study offers insights into the relationship between fear and amygdala activation. van Well *et al*. (2012) found that only subjects who exhibited differential fear-potentiated startle (FPS) responses to the CS+ vs. the CS− had higher CS+- than CS−-evoked amygdala BOLD signals. Subjects that did not exhibit differential FPS did not show differential amygdala activity, even when they showed normal US expectancy (i.e. they could correctly predict which CS would be followed by shock). Thus, conditioning, as indexed through correctly learning the stimulus contingencies, is not sufficient to elicit differential amygdala BOLD signals.

While van Well *et al*. (2012) were the first to measure FPS during human fMRI, many previous studies have used the skin conductance response (SCR), an autonomic measure of arousal, to index fear conditioning (LaBar *et al*., 1998; Phelps *et al*., 2004; Knight *et al*., 2005; Carter *et al*., 2006; Cheng *et al*., 2006, 2007). A robust finding from these studies is the positive correlation between differential CS+/CS− SCRs and differential amygdala BOLD signals. This is consistent with our study, which found a strong correlation between differential freezing responses and differential BLA T_O2_ signals. If subjects (rodent or human) do not show differential fear responses (as indexed by FPS, freezing or SCRs), it is unlikely that they will show differential amygdala activation. Thus, the levels of fear evoked by the CS+ compared with the CS− may be the critical determinant of differential amygdala activation.

### HPC T_O2_ signals discriminate between the CS+ and CS−

The precise role of the rodent HPC in fear conditioning remains debated. Early studies suggested that the HPC was required for contextual but not discrete cue conditioning (Selden *et al*., 1991; Phillips & LeDoux, 1992). However, both dHPC and vHPC lesions can reduce freezing to discrete cues under some circumstances (Richmond *et al*., 1999; Maren & Holt, 2004; Maren, 2008; Quinn *et al*., 2008; Zelikowsky *et al*., 2012).

The present data show that both dHPC and vHPC exhibit differential T_O2_ responses to discrete auditory cues. HPC T_O2_ signals discriminated between CS+ and CS− trials, both during training (vHPC) and during extinction (vHPC and dHPC). Moreover, HPC T_O2_ signals were markedly different from BLA T_O2_ signals, which is important because it shows that T_O2_ signals do not simply reflect general changes due to freezing behavior or the systemic physiological changes that accompany fear conditioning (e.g. heart rate, blood pressure, blood flow, etc.). Notably, there is good concordance between our vHPC T_O2_ data and anterior HPC BOLD signals recorded during human fear conditioning in terms of the timing and shape of the response (Alvarez *et al*., 2008; note that anterior HPC is the primate equivalent of rodent vHPC). In particular, in both humans and rodents the difference between CS+- and CS−-evoked signals is transient (i.e. the CS+ response is greater than the CS− response, but only during the first few seconds of stimulus presentation). Thus, differences may not be detected in the HPC when the BOLD contrast is based on analysis of longer durations.

Another notable finding is that CS+-evoked signals were significantly lower than CS−-evoked signals in both HPC subregions during extinction (i.e. there was a CS− > CS+ contrast). We do not know why the CS+-evoked HPC T_O2_ response is negative (compared with both the CS− and the pre-CS baseline), but recent evidence suggests that negative BOLD signals are associated with suppression of neuronal activity (Boorman *et al*., 2010). Exactly why the CS+, and not the CS−, is associated with this negative HPC response is not immediately obvious and requires further investigation. Nevertheless, higher CS−- than CS+-evoked HPC BOLD signals have also been reported during extinction in humans (Phelps *et al*., 2004). Thus, our HPC findings during extinction are consistent with human fMRI data.

### Using T_O2_ amperometry as a translational tool for BOLD fMRI

The present study is the first to employ time-resolved hemodynamic measurements in freely-moving rats, but previous studies have measured hemodynamic activity in rats after fear conditioning (LeDoux *et al*., 1983; Holschneider *et al*., 2006). For example, using cerebral blood flow (CBF)-autoradiography, Holschneider and colleagues found significantly increased CBF in the lateral amygdala in a fear-conditioned compared with a tone-alone group, but they also found significantly decreased CBF in the anterior BLA and central amygdala (Holschneider *et al*., 2006). While autoradiographic techniques can image the whole brain, their disadvantage is that they have no temporal resolution and offer only a snapshot of CBF changes in the brief (<1 min) period before the animal is killed. As a result, autoradiographic approaches are incompatible with the types of discriminative fear-conditioning designs used in human fMRI and the present study. In contrast, the T_O2_ approach used in the present study offers a high temporal resolution signal for studying hemodynamic changes associated with behaviour.

There are strong theoretical and empirical reasons for arguing that T_O2_ is closely related to BOLD (Zheng *et al*., 2002; Logothetis, 2007; Lowry *et al*., 2010). Nevertheless, there are several methodological differences between our approach and human fMRI studies of fear conditioning, including the species and the higher temporal and spatial resolution (but limited spatial sampling) of T_O2_ compared with BOLD. Importantly, however, our data are consistent with a subset of human fMRI studies that share certain key design features with our study, suggesting that these methodological differences are not critical.

Moreover, the T_O2_ approach utilized here is applicable to many other areas of neuroscience. Most of our knowledge of the cellular and molecular mechanisms that underlie behavior comes from animal experiments that for ethical and practical reasons are impossible in humans. Thus, animal models offer the best hope for understanding neuronal mechanisms underlying the dysfunctional brain states associated with neurological and psychiatric disorders. Techniques that facilitate comparisons between animal and human research are therefore essential if basic research is to translate to clinical benefits. One of the key advantages of our T_O2_ approach is that it can be combined with invasive methods, such as genetic modification, brain lesions, local drug infusions and electrophysiology; in other words, the techniques that allow us to uncover neuronal mechanisms. Thus, tissue oxygen amperometry has the potential to improve the translation between rodent models and human neuroimaging.

## Conclusion

To conclude, in rats, hemodynamic responses in the BLA, dHPC and vHPV can detect the differential patterns of neuronal activity evoked by threatening vs. neutral stimuli.

## Abbreviations

A–P: anterior–posterior
BLA: basolateral amygdala
BOLD: blood oxygen level-dependent
CBF: cerebral blood flow
CPE: carbon paste electrode
CS: conditioned stimulus
dHPC: dorsal hippocampal
D–V: dorsal–ventral
fMRI: functional magnetic resonance imaging
FPS: fear-potentiated startle
HPC: hippocampus
M–L: medial–lateral
SCR: skin conductance response
SME: simple main effects
US: unconditioned stimulus
vHPC: ventral hippocampal

## Supporting Information

**Fig. S1.** Behavioral (A) and tissue oxygen (T_O2_) responses in the basolateral amygdala (BLA; B), dorsal hippocampus (dHPC; C) and ventral hippocampus (vHPC; D) during pre-exposure, training and extinction of Pavlovian fear conditioning. Columns 1–4 show mean responses to the five CS− and five CS+ presentations during each day of phase I (pre-exposure, training I, training II and extinction, respectively). Columns 5 and 6 show responses for the first and last CS+ and CS− trials of extinction (i.e. column 5: CS−1 vs. CS+1; column 6: CS−5 vs. CS+5). (A) Mean (±SEM) percentage freezing during the 30 s of the CS− (blue bars) or CS+ (red bars). (B–D) Mean (±SEM) T_O2_ change from baseline (ΔT_O2_) during the 30 s of CS− (blue traces) and CS+ (red traces) in 2-s timebins. n.s., no significant difference in paired *t*-test, or no main effect of CS or CS × timebin interaction; *significant difference in paired *t*-test (*P <* 0.05) or main effect of CS (*P <* 0.05); ^#^CS × timebin interaction (*P <* 0.05).

**Fig. S2.** Freezing responses to the CS− (blue) and CS+ (red) during the last five trials of training. There was no evidence of discriminative freezing (i.e. higher freezing during CS+ trials) during any of the training trials.
