# Supplementary material for: Hemodynamic responses in amygdala and hippocampus distinguish between aversive and neutral cues during Pavlovian fear conditioning in behaving rats
Source: Eur J Neurosci. 2012 Nov 22;37(3):498–507. doi: 10.1111/ejn.12057 (PMC3638322; doi:10.1111/ejn.12057)
Supplement: Supplementary file 1 [file ejn0037-0498-SD1.pdf]

McHugh et al. “Hemodynamic...”
Supplementary Figure 1

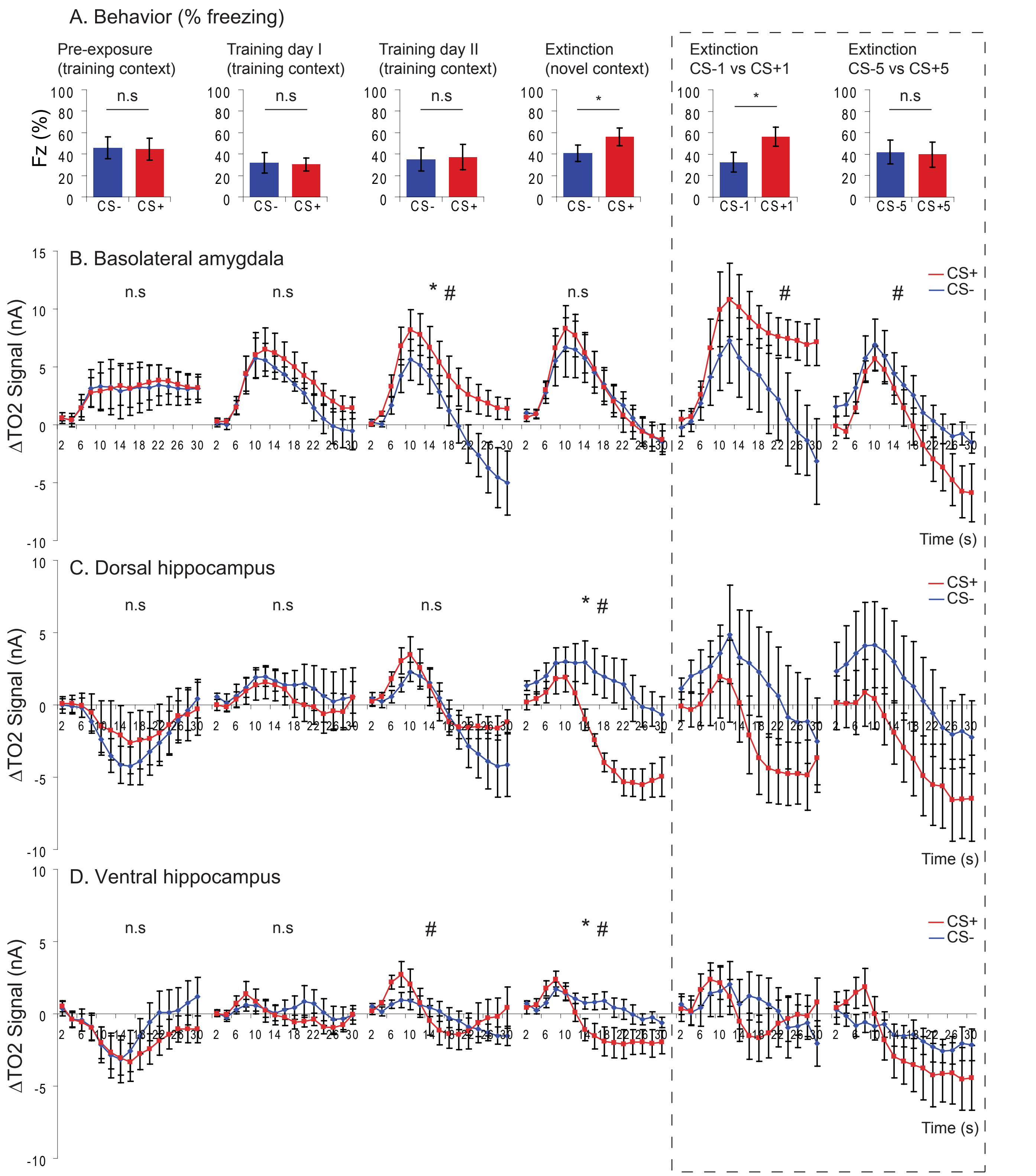

Supplementary Figure 1. Behavioral (A) and tissue oxygen (TO2) responses in the basolateral amygdala (B), dorsal hippocampus (C), and ventral hippocampus (D) during pre-exposure, training, and extinction of Pavlovian fear conditioning. Columns 1-4 show mean responses to the five CS- and five CS+ presentations during each day of phase I (pre-exposure, training I, training II, and extinction, respectively). Columns 5 and 6 show responses for the first and last CS+ and CS- trials of extinction (i.e. column 5: CS-1 versus CS+1; column 6: CS-5 versus CS+5). A. Mean (+/- SEM) percentage freezing during the 30s of the CS- (blue bars) or CS+ (red bars). B-D. Mean (+/- SEM) TO2 change from baseline (ΔTO2) during the 30s of CS- (blue traces) and CS+ (red traces) in 2s timebins. n.s. = no significant difference in paired t-test, or no main effect of CS or CS × timebin interaction, *significant difference in paired t-test ($P < 0.05$) or main effect of CS ($P < 0.05$), #CS × timebin interaction ($P < 0.05$).

McHugh et al. "Hemodynamic..."
Supplementary Figure 2

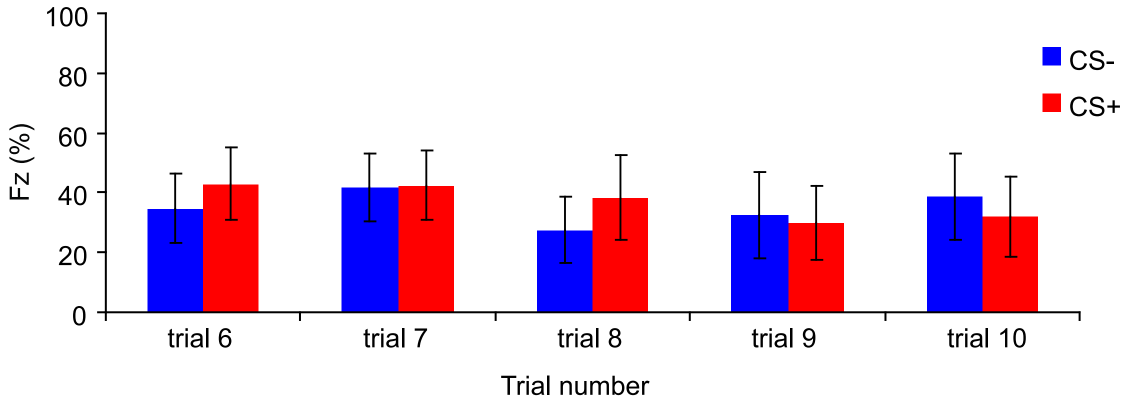

Supplementary Figure 2. Freezing responses to the CS- (blue) and CS+ (red) during the last 5 trials of training. There was no evidence of discriminative freezing (i.e. higher freezing during CS+ trials) during any of the training trials.
