# Supplementary figures and images for: Hemodynamic responses in amygdala and hippocampus distinguish between aversive and neutral cues during Pavlovian fear conditioning in behaving rats
Source: Eur J Neurosci. 2012 Nov 22;37(3):498–507. doi: 10.1111/ejn.12057 (PMC3638322; doi:10.1111/ejn.12057)

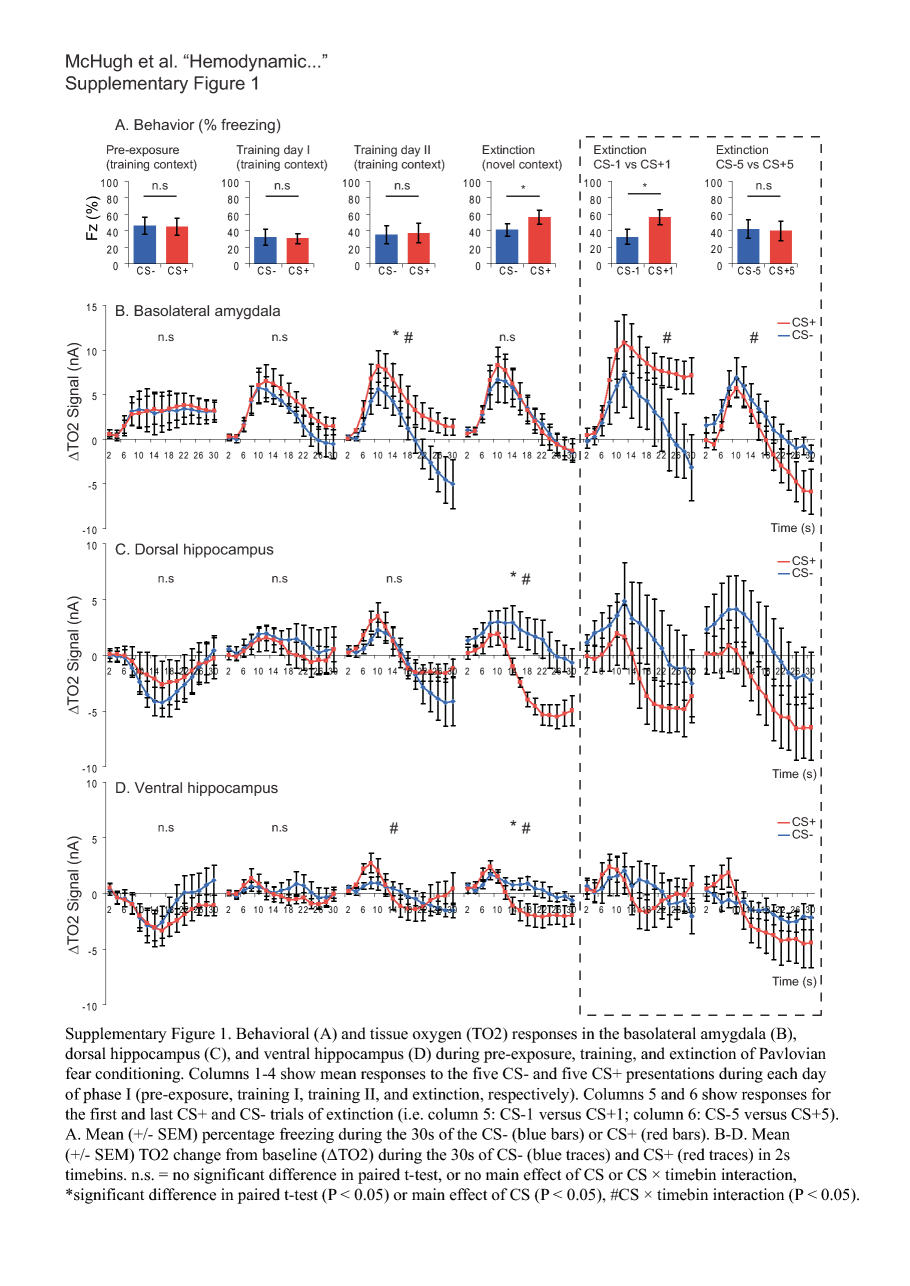

Supplement: Supplementary file 2 [file ejn0037-0498-SD2.png]
